# Perioperative switching to lemborexant for prevention of delirium in older cancer patients with insomnia taking GABA_A_ receptor agonists: a retrospective study

**DOI:** 10.1007/s00520-025-10197-2

**Published:** 2025-12-10

**Authors:** Tatsuto Terada, Takatoshi Hirayama, Ryoichi Sadahiro, Saho Wada, Junji Yamaguchi, Eri Nishikawa, Rika Nakahara, Shinsuke Washizuka, Hiromichi Matsuoka

**Affiliations:** 1 Department of Psycho-Oncology, National Cancer Center Hospital, Tokyo, Japan; 2 Department of Psychiatry, Shinshu University School of Medicine, Matsumoto, Japan; 3 Kokoro Support Clinic, Tokyo, Japan

**Keywords:** Postoperative delirium, Delirium prevention, GABA_A_ receptor agonists, Insomnia, Lemborexant

## Abstract

**Purpose:**

Postoperative delirium (POD) occurs in about one-third of patients and the incidence increases with age. The rate of missed delirium is 60%–70%, and there has recently been a shift from early detection and treatment to preventive strategies to reduce risk. While GABA_A_ receptor agonists (GRAs) are a risk factor for delirium, discontinuation may also worsen delirium through withdrawal or worsening of insomnia. This study aimed to evaluate the effect of switching from daily preoperative GRA therapy to lemborexant monotherapy on the incidence of postoperative delirium.

**Methods:**

A retrospective study was conducted in cancer patients aged ≥ 75 years who visited the Department of Psycho-Oncology at the National Cancer Center Hospital in Japan and were taking a GRA daily for insomnia before surgery under general anesthesia. Delirium was screened at least once daily with the Nursing Delirium Screening Scale on postoperative days 0–6 (positive if ≥ 2). We used a two-group intention-to-treat (ITT) framework, classifying patients according to a psycho-oncologist–verified preoperative switch to lemborexant.

**Results:**

Fifty patients satisfied eligibility. POD occurred in 1/17 (5.9%) in the Switch group and 15/33 (45.5%) in the Continue group; RD −39.6 percentage points (95% CI −59.9 to −19.2), RR 0.13 (95% CI 0.02–0.90), OR 0.08 (95% CI 0.01–0.63), *p* = 0.005.

**Conclusion:**

A preoperative switch policy to lemborexant monotherapy was associated with a lower observed risk of POD. Given the observational, unadjusted design, these associations are hypothesis-generating and cannot establish causality.

**Supplementary information:**

The online version contains supplementary material available at 10.1007/s00520-025-10197-2.

## Introduction

Delirium is associated with longer hospital stays, increased mortality, and the development of dementia [1]. Among cancer patients, delirium is estimated to affect 40% of hospitalized patients requiring palliative care [2] and 90% of patients at the end of life [3]. Furthermore, the incidence of delirium is thought to increase with aging. Japan has one of the most aged populations worldwide, and statistics from the Ministry of Health, Labour and Welfare in 2020 indicate that more than half of all hospitalized patients are aged ≥ 75 years old [4], with this number likely to increase in the future. Thus, Japan may provide a global model for examining anti-delirium strategies for elderly patients with cancer.

Management of delirium primarily involves treatment of the underlying disease, but drug therapy may worsen delirium and the prognosis [5]. Delirium is also frequently missed in clinical evaluations, and it is a concern that the rate of missed cases, which was 55%–70% in 2000–2001 and 60% in 2015, has not significantly improved over time [6]. Preventive strategies are also important for reducing the risk of delirium [3].

Postoperative delirium (POD) is estimated to affect 10%–30% of patients [7, 9], and this rate can be as high as 50% in patients undergoing high-risk surgery [10]. One risk factor for POD is preoperative routine daily use of GABA_A_receptor agonists (GRAs) [11, 12]. However, more than 90% of clinicians, including psychiatrists, have experienced difficulties in GRA discontinuation [13], and reducing the risk of POD by GRA discontinuation is generally regarded as difficult. GRA discontinuation is also a risk factor for withdrawal delirium, and the feasibility of discontinuation itself should also be carefully considered. In addition, GRA discontinuation may worsen insomnia. Considering that disturbances of sleep–wake rhythm, including insomnia, are also risk factors for delirium [14], care must be taken to ensure that insomnia does not occur as a result of GRA discontinuation.

Based on these findings, GRA discontinuation and maintenance of sleep–wake rhythms to minimize the effects of withdrawal are necessary to reduce the risk of GRA-related POD. If sleep–wake rhythms can be maintained while switching from GRA to lemborexant, the risk of POD due to GRAs and insomnia may be reduced, but evidence in support of this hypothesis is lacking. Thus, the purpose of this study was to determine if switching from GRA to lemborexant reduces the incidence of POD in elderly patients with cancer.

## Methods

A retrospective chart review was conducted to evaluate the effect of switching from GRA to lemborexant on the incidence of POD. The study was conducted at the National Cancer Center Hospital (NCCH) in Tokyo, Japan. The inclusion criteria were as follows: (1) visited the Department of Psycho-Oncology for perioperative support from January 2021 to December 2023; (2) age ≥ 75 years; (3) received routine daily GRA therapy for insomnia; (4) assessed for delirium symptoms using the Nursing Delirium Screening Scale (Nu-DESC); and (5) scheduled for cancer surgery under general anesthesia. The exclusion criteria were as follows: (1) already participating in another clinical trial on delirium; (2) switched to any non-GRA other than lemborexant; (3) switched from GRA to Lemborexant < 18 days before the day of surgery; and (4) treatment with lemborexant in combination with a GRA.

Data were collected on background factors (age, sex, cancer type, alcohol consumption); Nu-DESC (maximum score once daily on postoperative days 0–6); Charlson Comorbidity Index (CCI); Mini-Mental State Examination-Japanese score’ diazepam-equivalent daily dose (DEDD) of preoperative GRA [15]; operative time; intensive care unit (ICU) admission; and status of GRA administration (Switch group: switched from GRA to lemborexant; Continue group: continued with GRA therapy). Cases in which a switch was intended but not sustained were kept in the Switch (lemborexant) group in order to reflect the initial switch policy, regardless of the regimen at surgery.

Chart reviews were performed by two psycho-oncologists (TT, TH). Consensus on the interpretation of the collected data was confirmed, and any conflicting opinions were discussed and decided upon on a case-by-case basis. The Nu-DESC assessments were performed at least once daily in the perioperative ward as part of routine nursing practice, and no patients were excluded for lack of Nu-DESC. Nu-DESC evaluates delirium symptoms on a 2-point scale for each of 5 items, with a total score of 10 points. Delirium was defined as a Nu-DESC score of ≥ 2 [16]. In cases for which a psycho-oncologist was co-consulted in the postoperative period, we performed a sensitivity analysis, using the psycho-oncologist’s diagnosis based on criteria from the Diagnostic and Statistical Manual of Mental Disorders, Fifth Edition [17] as the outcome. The observation period for the Nu-DESC was 7 days, with reference to a previous study on POD [18]. Typical withdrawal delirium [19] occurs 3–7 days after discontinuation and lasts 3–10 days; thus, up to 18 days may be required to reduce the effects of withdrawal delirium after GRA discontinuation. Alcohol consumption status was defined as whether or not the patient had consumed alcohol within 18 days of surgery (including the day of surgery) to exclude the effects of withdrawal. CCI scores (≥ 3 vs. < 3) were used descriptively to confirm comparable comorbidities between groups.

For the primary analysis, we adopted a two-group intention-to-treat (ITT) framework that reflects the preoperative treatment policy. The exposure classification required verification of a psycho-oncologist’s prescription for lemborexant monotherapy in the medical record at the time of the psycho-oncology consultation. Patients with a verified prescription were analyzed in the Switch (lemborexant) group regardless of subsequent deviations (e.g., temporary resumption of any GRA, perioperative pro re nata GRAs, or “switching failure” in which the initial plan was not sustained through the day of surgery). Patients without such verification were assigned to the Continue group, irrespective of dose adjustments within the GABA_A_ class. To ensure stable exposure, decisions verified < 18 days before surgery were excluded from the ITT set. This policy-based ITT strategy targets the treatment-policy effect of a preoperative switch to lemborexant monotherapy on the 7-day postoperative risk of delirium.

Treatment decisions were made case-by-case by psychiatrists specializing in psycho-oncology. Exposure was assigned at the time of the psycho-oncology consultation based on verification of a prescription for lemborexant monotherapy. The specific intent or rationale for individual medication changes was not systematically recorded, which must be considered when interpreting potential bias related to baseline hypnotic exposure.

The study was approved by our institutional review board (approval number: 2018–130). The requirement for informed consent was waived due to the retrospective design. Opt-out information was published on the NCCH website.

### Statistical analysis

Categorical variables are presented as percentages and were analyzed by the chi-square (χ^2^) test or Fisher’s exact test. Continuous variables are presented as the median and interquartile range (IQR) and were analyzed by the Wilcoxon rank-sum test. We report absolute risks, risk differences (RD), risk ratios (RR), and odds ratios (OR) with 95% confidence intervals (95% CI); *p*-values are presented as secondary.

The approach to confounding was as follows. Because of the small sample and sparse events, the primary analyses were prespecified as unadjusted; we therefore do not infer causality from observed associations. Candidate confounders were defined a priori as variables plausibly influencing both treatment selection (preoperative switch to lemborexant monotherapy vs continuation of GRA) and postoperative delirium risk. Domains included preoperative GRA exposure (dose/duration; DEDD), alcohol use, baseline cognition, centrally acting co-medications and anticholinergic burden, age/frailty/performance status, comorbidity burden, and surgical factors (site, operative time). Variable-level data availability (measured/measured by proxy/partial/unavailable) is summarized in Supplementary Table S1.

Stratified sensitivity analyses were carried out with the following definitions. Exploratory strata were prespecified as MMSE-J high (≥ 24) vs low (< 24) [20], DEDD low vs high by the cohort median preoperative dose, and alcohol use yes (any alcohol consumption within the 18 days prior to surgery [19], including the day of surgery) vs no. Within each stratum, we report absolute risks, RD, and RR/OR with 95% CIs using exact/binomial methods appropriate for small samples; when a zero cell occurred, we prioritized RD with exact CIs and treated ratio measures as non-estimable.

These tests were used to compare the characteristics of the Switch and Continue groups. All statistical analyses were performed using JMP® Student Edition 19.0.1 (SAS Institute Inc., Cary, NC), with significance set at *p* < 0.05.

## Results

**Fig. 1:**
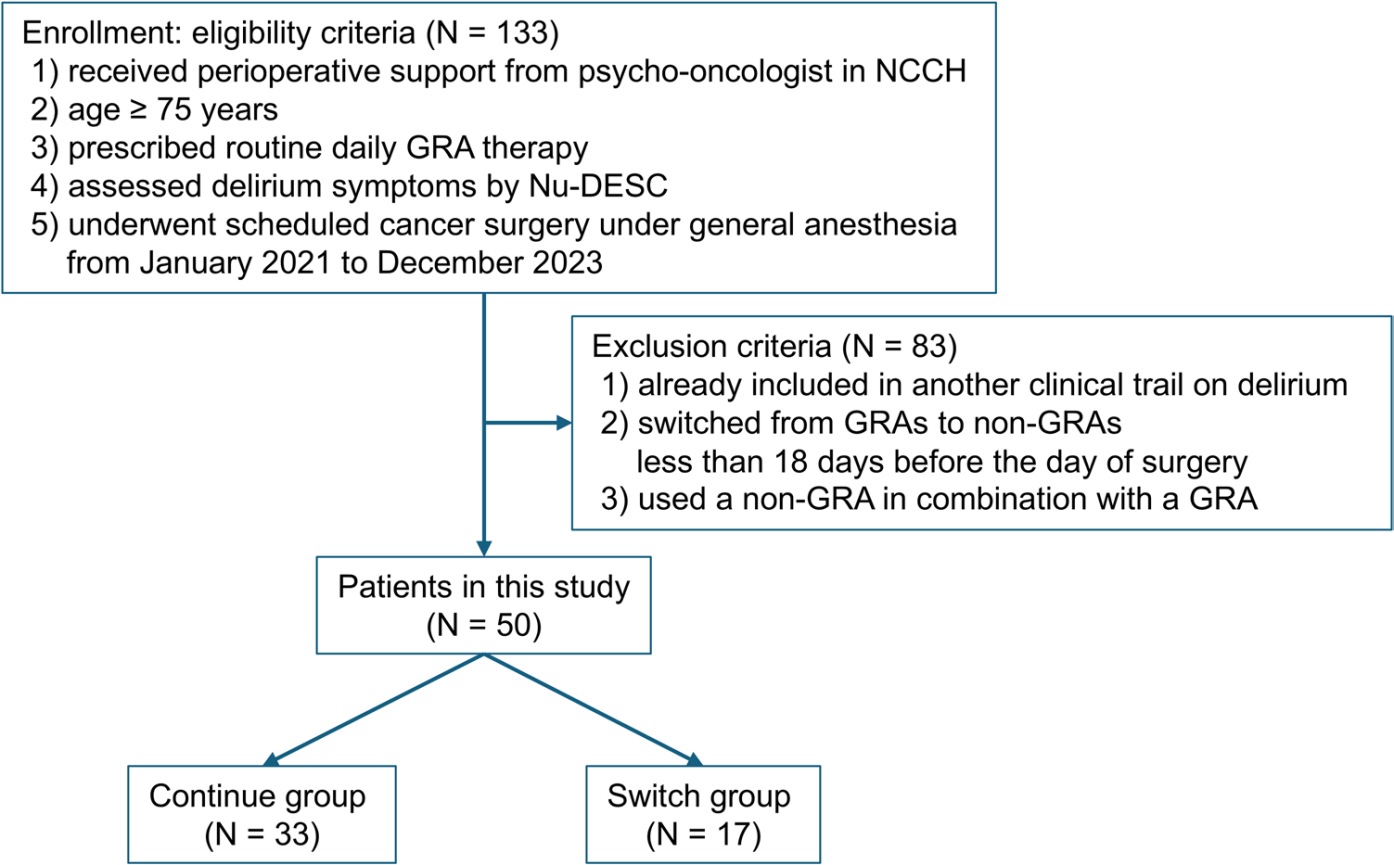
Flowchart of patients in the study

A total of 50 patients (19 females) with cancer satisfied the eligibility criteria (Fig. 1). Age ranged from 75 to 87 years (median 78.5; IQR, 76, 81) (Table 1) and the DEDD ranged from 0.625 to 21 mg (median 5; IQR, 2.5, 7.5). POD developed in 16 patients (32.0%).

**Table 1:** Baseline characteristics

Item	All (*n* = 50)	Continue (*n* = 33)	Switch (*n* = 17)	*p*
Age, median (IQR)	78.5 (76, 81)	78 (76, 81)	79 (77, 83.5)	0.339
Male sex, *n* (%)	31 (62.0)	22 (66.7)	9 (52.9)	0.373
Cancer type, *n* (%)				0.065
Head and neck	17 (34.0)	13 (39.4)	4 (23.5)	
Esophageal	3 (6.0)	1 (3.0)	2 (11.8)	
Lung	6 (12.0)	6 (18.2)	0 (0)	
Gastrointestinal	9 (18.0)	3 (9.1)	6 (35.3)	
Hepatobiliary pancreatic	8 (16.0)	4 (12.1)	4 (23.5)	
Skin	1 (2.0)	1 (3.0)	0 (0)	
Other	6 (12.0)	5 (15.2)	1 (5.9)	
Ongoing alcohol use within 18 days pre-op^a^, *n* (%)	7 (14.0)	7 (21.2)	0 (0)	0.080
CCI ≥ 3, *n* (%)	26 (50.0)	19 (57.6)	6 (35.3)	0.232
MMSE-J^b^, median (IQR)	28 (25.25, 29)	28 (25, 29)	28 (26.25, 29.75)	
DEDD^c^, median (IQR)	5 (2.5, 7.5)	5 (2.5, 8.75)	5 (2.5, 5)	0.093
Operative time, median (IQR)	206.5 (112, 387.5)	170 (97, 396)	262 (136.5, 395)	0.334
HADS-A^d^, median (IQR)	4 (1, 9)	4 (0.75, 10.5)	4 (1, 7.5)	
HADS-D^d^, median (IQR)	5 (1, 10)	7.5 (1.5, 10)	5 (0.5, 8.5)	
ICU admission, n (%)	20 (40.0)	13 (39.4)	7 (41.2)	1.000

*IQR* interquartile range, *CCI* Charlson Comorbidity Index, *DEDD* diazepam-equivalent daily dose of preoperative GABA_A_ receptor agonists

^a^Alcohol use denotes any alcohol consumption within the 18 days prior to surgery, including the day of surgery

^b^MMSE-J was partially recorded (missing *n* = 10)

^c^DEDD strata were defined by the cohort median of preoperative diazepam-equivalent daily dose

^d^HADS was partially recorded (missing *n* = 27)

In the two-group ITT analysis, 1/17 (5.9%) in the Switch (lemborexant) group developed POD compared with 15/33 (45.5%) in the Continue group; RD = −39.6 pp (95% CI −59.9 to −19.2), RR = 0.13 (95% CI 0.02–0.90), OR = 0.08 (95% CI 0.01–0.63), *p* = 0.005. In a subgroup with psycho-oncologist assessment (*n* = 38), delirium occurred in 2/12 (16.7%) in the Switch group compared with 14/26 (53.9%) in the Continue group (*p* = 0.040). Using Nu-DESC ≥ 2, sensitivity and specificity were 81.3% and 86.4%, respectively.

Definitions for strata in stratified analyses are provided in the Methods. In available-case analyses by cognition, among patients with high MMSE-J scores, delirium occurred in 1/15 (6.7%) in the Switch group versus 7/21 (33.3%) in the Continue group (RD −0.267, 95% CI −0.50 to −0.029; RR 0.20, 95% CI 0.027 to 1.459; OR 0.143, 95% CI 0.015 to 1.318). The low MMSE-J stratum contained only 4 patients overall; estimates were unstable and are not emphasized.

By GRA exposure, the association favored Switch in both strata: low DEDD, delirium occurred in 1/14 (7.1%) in the Switch group versus 7/18 (38.9%) in the Continue group (RD −0.317, 95% CI −0.58 to −0.055; RR 0.184, 95% CI 0.026 to 1.324; OR 0.121, 95% CI 0.013 to 1.140); high DEDD, delirium occurred in 0/3 (0%) in the Switch group versus 8/15 (53.3%) in the Continue group (RD −0.533, 95% CI −0.785 to −0.280) with ratio measures not estimable due to a zero cell in the Switch group.

By alcohol use, ongoing alcohol use was observed only in the Continue group; among non-drinkers, delirium occurred in 1/17 (5.9%) in the Switch group versus 13/26 (50.0%) in the Continue group (RD −0.441, 95% CI −0.664 to −0.219; RR 0.117, 95% CI 0.017 to 0.819; OR 0.063, 95% CI 0.007 to 0.543).

Baseline characteristics such as age and sex were generally similar between groups; however, the Continue group tended to have higher alcohol consumption (*p* = 0.080) and preoperative DEDD (*p* = 0.093). ICU admission and operative time did not differ meaningfully. Details of the lemborexant regimen in the Switch group are summarized in Table 2.

**Table 2:** Lemborexant regimen in the Switch group

Drug	Number of cases	Number of cases with delirium
Lemborexant 2.5 mg	7	0
Lemborexant 5 mg	10	1

Switching fidelity and adverse events were examined in the Switch group. Three cases met the definition of “switching failure” (Table 3). Two patients resumed GRA therapy without any increase in the maximum preoperative dose [21]. One patient receiving lemborexant 2.5 mg monotherapy reported palpitations and nightmares and discontinued the drug without medical consultation; therefore, potential withdrawal symptoms could not be evaluated. No serious adverse events were recorded, and no dose escalations occurred among those who resumed GRA therapy. While these observations suggest acceptable tolerability in this small cohort of older patients (≥ 5 years), robust safety inferences are not possible because of the small sample size.

**Table 3:** Details of cases with switching failure within the Switch group (two-group ITT)

Case	Choice of non-GRA therapy	Reason for failure
1	Lemborexant 2.5 mg	Ineffectiveness
2	Lemborexant 2.5 mg	Palpitations and nightmares
3	Lemborexant 5 mg	Ineffectiveness

Switching failure denotes an attempted preoperative switch that was not sustained; under the ITT policy, these cases remained in the Switch group

## Discussion

Our findings suggest that a preoperative switch policy to lemborexant monotherapy might be associated with a lower risk of POD in older cancer patients who previously used daily GRAs. Delirium is the deterioration of brain function caused by a multifactorial process [22] and routine preoperative use of GRA is considered to be a risk factor for delirium. This study illustrated the negative effects of GRAs, demonstrated the possibility of replacing GRA therapy with lemborexant, which has been considered difficult [13], and showed that switching from GRA to lemborexant was associated with a lower risk of postoperative GRA-related delirium.

This study has several limitations, including its retrospective design, small sample size, and confounding by indication. Therefore, these results should be interpreted as hypothesis-generating. Second, outcome ascertainment relied on the Nu-DESC, which may under-detect hypoactive delirium when a threshold of ≥ 2 is used. In a subgroup analysis with psycho-oncologist assessment (*n* = 38), delirium was found to have occurred in 2/12 (16.7%) in the Switch group and 14/26 (53.9%) in the Continue group (*p*= 0.040). Using Nu-DESC ≥ 2, sensitivity and specificity were 81.3% and 86.4%, respectively. Prior research indicates that the Nu-DESC assists in detecting symptoms of POD that nurses might otherwise miss [23]; likewise, in the present study, it appeared to be useful for identifying POD in older adults. Although we restricted inclusion to patients with documented Nu-DESC assessments, no cases were excluded due to missing Nu-DESC; therefore, we do not anticipate meaningful selection bias from this criterion. Residual and unmeasured confounding cannot be excluded (e.g., GRA dose and duration as well as baseline insomnia severity). Confounding and non-causal interpretation are other limitations that must be considered. This study did not adjust for confounding; therefore, findings are hypothesis-generating rather than causal. Confounders were considered a priori as variables plausibly affecting both treatment selection and delirium risk, including preoperative GRA exposure (dose/duration; DEDD); alcohol use; baseline cognition; psychiatric comorbidities (anxiety/depression); centrally acting co-medications (opioids, anticholinergics, gabapentinoids, sedative-hypnotics); anticholinergic burden; age/frailty/performance status; comorbidity burden; and surgical/anesthesia factors (type/site/operative time). In our cohort, DEDD was higher and alcohol use more frequent in the Continue group, which is consistent with confounding by indication, which could bias in favor of the Switch group (i.e., exaggerate the observed benefit of switching). Cognition (MMSE-J) was only partially captured, which may reinforce this bias if there is imbalance between the groups. Surgical/anesthesia factors were measured (no emergency cases) and operative time did not differ materially between the groups, suggesting limited confounding from surgical invasiveness. Variable-level data availability is detailed in Supplementary Table S1. In stratified sensitivity analyses, the association consistently favored the Switch group across high MMSE-J, low DEDD, and non-drinker strata, with large absolute differences despite limited precision in some cells; the high DEDD stratum showed a similar direction but was underpowered. These findings support a hypothesis-generating interpretation while acknowledging that baseline imbalances (e.g., higher DEDD and more alcohol use in the Continue group) could bias in favor of the Switch group. Third, clinicians may have refrained from switching in some patients with higher GRA doses or longer durations, prominent preoperative anxiety with insomnia, or concerns about psychological or physical dependence—for example, to mitigate the risk of withdrawal-related delirium or clinical destabilization. Consistent with this possibility, the Continue group had greater preoperative GRA exposure (DEDD) and an imbalance in alcohol use at baseline. Taken together, these observations suggest confounding by indication. This confounding by indication might bias unadjusted comparisons despite our decision-based ITT approach; thus, it is not possible to infer causality. Fourth, the incidence of POD may have included some cases of withdrawal delirium in the present study; it is unclear whether the delirium observed was purely postoperative or related to the discontinuation of GRA after surgery. However, the extent of delirium due to GRA withdrawal is difficult to predict. Finally, several a priori confounder domains were only partially captured (e.g., baseline cognition, psychiatric comorbidity, centrally acting co-medications/anticholinergic burden); these were therefore listed for transparency but were not analyzed, leaving potential residual and unmeasured confounding (see Supplementary Table S1). Post-decision deviations (i.e., switching failure may also introduce exposure misclassification); our policy-based ITT assigned exposure at the time of the preoperative decision.

In conclusion, our findings are hypothesis-generating and support further prospective evaluation of a preoperative switch to lemborexant monotherapy in older cancer patients using daily GRAs. Although this was a retrospective study based on a small number of cases in a single center, the data reported here can serve as a basis for designing multicenter studies. Despite the limitations, our findings suggest that switching from GRA to lemborexant in patients undergoing scheduled surgery was associated with a lower risk of POD. We plan to conduct a prospective study to explore the efficacy of switching from GRA to lemborexant in the prevention of POD. Studies are also needed to examine the response in unscheduled surgeries that do not allow sufficient time for this switch.

## Supplementary information

Below is the link to the electronic supplementary material.

Supplementary file 1 (PDF 140 KB)

## Data Availability

The data that support the findings of this study are available from the corresponding author (HM) upon reasonable request.
