# Supplementary material for: Perioperative switching to lemborexant for prevention of delirium in older cancer patients with insomnia taking GABAA receptor agonists: a retrospective study
Source: Support Care Cancer. 2025 Dec 10;34(1):22. doi: 10.1007/s00520-025-10197-2 (PMC12696088; doi:10.1007/s00520-025-10197-2)
Supplement: Supplementary file 1 — (PDF 140 KB) [file 520_2025_10197_MOESM1_ESM.pdf]

Supportive Care in Cancer

Supplementary Information

Perioperative switching to lemborexant for prevention of delirium in older cancer patients with insomnia taking $GABA_A$ receptor agonists: a retrospective study

Tatsuto Terada, MD, Takatoshi Hirayama, MD, Ryoichi Sadahiro, MD, PhD, Saho Wada, MD, PhD, Junji Yamaguchi, MD, Eri Nishikawa, MD, PhD, Rika Nakahara, MD, PhD, Shinsuke Washizuka, MD, PhD, and Hiromichi Matsuoka*, MD, PhD

*Correspondence to: Hiromichi Matsuoka, MD, PhD, National Cancer Center Hospital, 5-1-1 Tsukiji, Chuo-ku, Tokyo, 104-0045 Japan; E-mail: hiromima@ncc.go.jp

Contents

Supplementary Methods

Supplementary Table S1

Supplementary Methods

A priori confounder set and data availability

To enhance transparency about potential confounding, we specified a priori confounders as variables plausibly affecting both treatment selection (preoperative switch to lemborexant monotherapy vs continuation of $GABA_A$ receptor agonists) and the risk of postoperative delirium (POD) in older cancer surgical patients. Given the small sample size and sparse events, the primary analyses were prespecified as unadjusted; we therefore do not draw causal inferences from observed associations. Instead, we list the a priori confounders and their variable-level availability in this dataset to aid interpretation.

Supplementary Table S1 A priori confounders affecting both treatment selection and postoperative delirium, and their availability in the present study

Confounder domain	Causal rationale (treatment selection ↔ POD)	Availability in this study	Operationalization/notes
Age / frailty / performance status	Older/frailer patients have higher POD risk; may influence switching	Measured (age) / Unavailable (frailty/PS)	Exact age summarized in Table 1; frailty/PS not systematically captured.
Comorbidity burden (e.g., CCI)	Higher burden → higher POD risk; may affect treatment selection	Measured	CCI categories summarized in Table 1
Preoperative GRA exposure (dose/duration)	Higher exposure may favor continuation; linked to POD	Measured (proxy)	DEDD summarized in Table 1; duration not captured. DEDD stratified (low/high) in sensitivity analyses.
Alcohol use / alcohol use disorder (AUD)	May influence treatment selection; POD risk factor	Measured	Alcohol use summarized in Table 1; stratified (any alcohol consumption within 18 days pre-op / none) in sensitivity analyses; none diagnosed with AUD.
Baseline cognition	Strong POD determinant; may deter switching	Partial	MMSE-J summarized in Table 1; stratified (high (≥ 24) / low (< 24)) in sensitivity analyses; 10 cases missing.
Psychiatric comorbidity (anxiety/depression)	Affects treatment selection and POD	Partial	HADS summarized in Table 1; 27 cases missing.
Centrally acting co-medications and anticholinergic burden	Increase POD risk; may constrain switching	Partial	Partially captured from MAR; used for exclusion criteria of "switched to any non-GRA other than lemborexant"
Surgical factors (site, operative time)	Core POD drivers	Measured	Site/operative time summarized in Table 1
Sensory impairment (vision/hearing)	Predisposing factors for delirium; may deter switching	Unavailable	Nursing records reviewed; unclear; not considered.
Pain severity	May precipitate delirium; may deter switching	Unavailable	Nursing records reviewed; unclear; not considered.

Availability categories: measured (direct variable present); measured (proxy) (surrogate such as DEDD for benzodiazepine exposure); partial (incompletely considered in the present study); unavailable (not considered in the present study).

DEDD, diazepam-equivalent daily dose; CCI, Charlson Comorbidity Index; GRA, $GABA_A$ receptor agonists; PS, performance status; MAR, medication administration record; POD, postoperative delirium; MMSE-J, Mini-Mental State Examination—Japanese.
